# A rare case of pigmented villonodular synovitis after unicompartmental knee replacement: a case report

**DOI:** 10.1186/1757-1626-2-9076

**Published:** 2009-11-23

**Authors:** Paraskumar Mohanlal, Dilip Pillai, Sunil Jain

**Affiliations:** 1 Department of Trauma and Orthopaedics, Medway Maritime Hospital, Gillingham, Kent, ME7 5NY, UK

## Abstract

Pigmented villonodular synovitis is a benign proliferative disease involving the synovium. Pigmented villonodular synovitis is rare after replacement arthroplasty and has not been recognised and reported as a cause of failure of unicompartmental knee replacement in the literature.

## Introduction

Pigmented villonodular synovitis (PVNS) is a proliferative disease affecting the synovial joints resulting in villous or nodular changes in the synovial tissue, large effusions and bony erosions [1]. PVNS after replacement arthroplasty is rare and to our knowledge, only one such case has been reported in the literature [2]. We report a case of PVNS after unicompartmental knee replacement (UKR).

## Case Report

A 69-year-old retired Caucasian gentleman with a BMI of 30, presented with one year history of activity related anteromedial knee pain without any mechanical symptoms. He previously underwent medial unicompartmental knee replacement 4 years ago and was asymptomatic till his symptoms started insidiously a year ago. He suffers from hypertension and does not have any other co-morbidities. He is a non-smoker and drinks alcohol occasionally.

**Figure 1:**
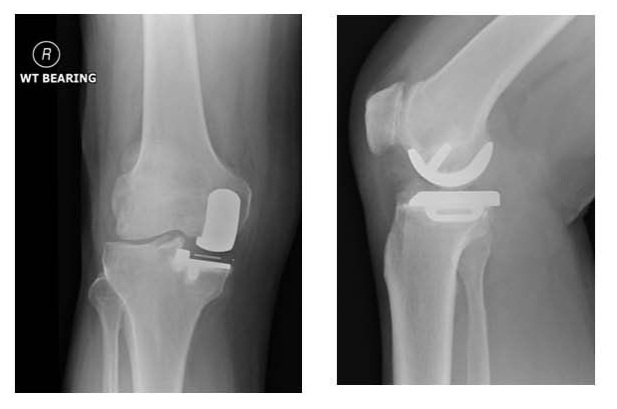
**Pre-operative anteroposterior and lateral radiographs of the patient showing medial unicompartmental prosthesis in-situ**.

Clinically he had a well healed surgical scar over the anteromedial aspect of the knee, moderate effusion, mild tenderness over the medial tibial plateau, and no signs of instability with the last 10 degrees of flexion restricted. Radiographs showed good alignment of the medial unicompartmental prosthesis with no evidence of loosening (Figure 1). Blood investigations including inflammatory parameters were within normal limits and bone scan including an Indium White Cell scan ruled out evidence of infection or loosening.

**Figure 2:**
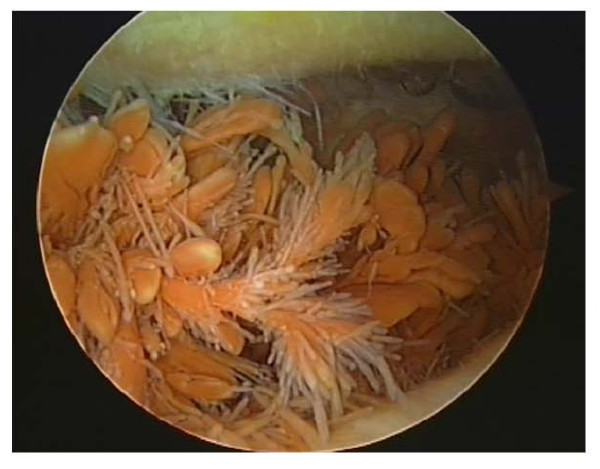
**Intra-operative arthroscopic pictures demonstrating synovial proliferation suggestive of pigmented villonodular synovitis**.

**Figure 3:**
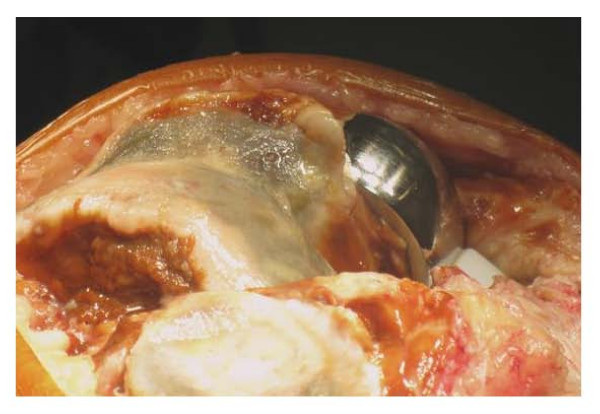
**Intra-operative photographs taken during revision to total knee replacement demonstrating pigmentation and arthritis involving the lateral femoral condyle**.

**Figure 4:**
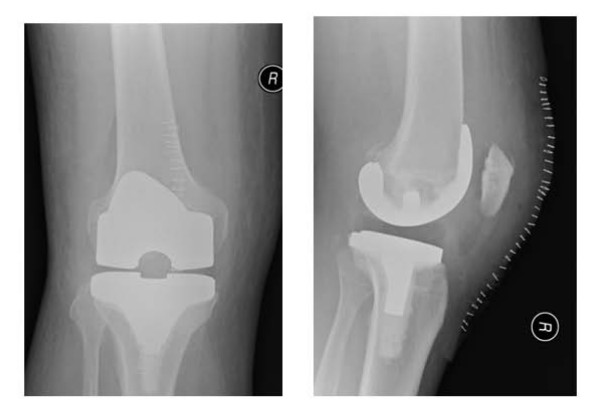
**Post-operative anteroposterior and lateral radiographs of the patient after revision to a total knee replacement**.

He underwent arthroscopy of the knee which showed evidence of pigmented proliferation with villous appearance of the synovial tissue suggestive of PVNS (Figure 2). Arthroscopic debridement with synovectomy was done and histological analysis of the synovial tissue confirmed the diagnosis of pigmented villonodular synovitis. He underwent revision to a total knee replacement and is recovering well (Figure 3 and 4).

## Discussion

PVNS is a benign proliferative disorder of the synovium of unknown etiology [1]. It can affect bursae, tendon sheaths and joints and was first reported in 1941 [3,4]. PVNS can pose a diagnostic challenge. Studies have suggested that PVNS is diagnosed on an average 4.4 years after presentation [5]. The incidence is 1.8 per one million population and the knee is the commonest joint to be affected [6].

PVNS may be intra- or extraarticular and can present in the diffuse or nodular form with a tendency to bleed and erode bone [1]. Joint effusion can be out of proportion to the mild degree of pain and absence of bloody effusion does not rule out PVNS [1,7,8]. Radiologically there is bone preservation and relative lack of hypertrophic bone or spur formation in PVNS can help differentiate this condition from osteoarthritis [1,8]. The incidence of juxta-articular bone involvement in the knee is less and is postulated to be due to capacious knee capsule and large suprapatellar bursa providing space for tumour like growth of PVNS [1]. Histologically it has multinucleated giant cells with characteristic pigmentation due to intra- and extra cellular hemosiderin perhaps due to its tendency to bleed [1].

The suggested treatment for PVNS depends on the severity of joint involvement and age of the patient. The options are synovectomy, radiotherapy, arthrodesis and arthroplasty [1,9,10]. Our patient presented with symptoms of anteromedial knee pain without any instability symptoms. Radiographs did not show any signs of loosening of the prosthesis and PVNS was not initially considered in the differential diagnosis. However at arthroscopy, there was hypertrophy with villous appearance of the synovial tissue, associated with evidence of lateral compartment arthritis. Although synovectomy was performed, he continued to be symptomatic and hence revision to a total knee replacement was done. Post-operatively patient recovered well, and is mobilising well without any walking aids and full resolution of symptoms.

The main reasons for failure of unicompartmental knee replacement are infection, aseptic loosening, mal-alignment, other compartment degeneration, polyethylene wear and instability [11]. In our patient the symptoms were related to PVNS, which subsequently improved after revision to a total knee replacement.

This case highlights an unusual case of PVNS as a cause of failure of unicompartmental knee replacement. Although rare, we suggest considering PVNS in the differential diagnosis of patients presenting with knee pain after unicompartmental knee arthroplasty.

## Competing interests

The authors declare that they have no competing interests.

## Consent

Written informed consent was obtained from the patient for publication of this case report and accompanying images. A copy of the written consent is available for review by the Editor-in-Chief of this journal

## Authors' contributions

PM, DP and SJ have been involved in the treatment of the patient, literature search and manuscript preparation. All authors have read and approved the final manuscript.
